# Antiproliferative Activity of Plant Extracts Used Against Cancer in Traditional Medicine

**DOI:** 10.3797/scipharm.0912-11

**Published:** 2010-02-13

**Authors:** Wamidh H. Talib, Adel M. Mahasneh

**Affiliations:** Department of Biological Sciences, Faculty of Science, University of Jordan, Amman-11942, Jordan

**Keywords:** Anti-cancer activity, Medicinal plants, MTT and TUNEL assays, Traditional use

## Abstract

Forty four extracts from sixteen plants used traditionally as anticancer agents were evaluated *in vitro* for their antiproliferative activity against Hep-2, MCF-7, and Vero cell lines. Plants were fractionated using ethanol, methanol, chloroform, *n*-hexane, distilled water, and butanol. The antiproliferative activity was measured by MTT assay. TLC was used to identify active fractions. The apoptotic activity of active fractions was determined using TUNEL colorimetric assay. 20 of these extracts demonstrated significant antiproliferative activity against one or more of the cell lines. These extracts were prepared from *Ononis hirta*, *Inula viscosa*, *Salvia pinardi*, *Verbascum sinaiticum* and *Ononis sicula*. Methanol fractions of *Ononis hirta* (aerial parts) and *Inula viscosa* (flowers) were the most active fractions against MCF-7 cells with IC_50_ of 27.96 and 15.78 μg/ml respectively and they were less toxic against other cell lines. Other extracts showed lower activity against cancer cell lines. TLC analysis showed the presence of flavonoids and terpenoids in active plants while alkaloids were detected in *Ononis hirta* (aerial parts) extracts. *Ononis hirta* (aerial parts) and *Inula viscosa* (flowers) extracts exerted their antiproliferative activity by inducing apoptosis in cancer cell lines. Further studies are necessary for detailed chemical characterization and more extensive biological evaluation of the most active ingredients.

## Introduction

Plants are considered among the main sources of biologically active chemicals. It has been estimated that about 50% of the prescription products in Europe and USA are originating from natural products or their derivatives [1]. Out of the 250,000–500,000 plant species on earth, only 1–10% have been studied chemically and pharmacologically for their potential medicinal value [2]. In Middle East region 700 species of identified plants are known for their medicinal values [3]. In Jordan, out of the 2500 plant species recorded, more than 100 species are listed as endemic and of medicinal potential in folk medicine [4]. A comprehensive survey on practitioners and herbalists in Jordan indicated that more than 150 plant herbs still in use as traditional source of herbal medicine [5]. However, little is known about the possible medicinal application of these plants [6–8]. Plants like *Teucrium polium*, *Phagnalon rupstre*, and *Inula viscosa* are prescribed to alleviate diseases including tumor reduction, ulceration, diabetes, kidney stones, inflammations, rheumatism, and muscle relaxation [9, 10].

In spite of the recent domination of the synthetic chemistry as a method to discover and produce drugs, the potential of bioactive plants or their extracts to provide new and novel products for disease treatment and prevention is still enormous [11]. The antitumor area has the greatest impact of plant derived drugs, where drugs like vinblastine, vincristine, taxol, and camptothecin have improved the chemotherapy of some cancers [1]. Plants have an almost unlimited capacity to produce substances that attract researchers in the quest for new and novel chemotherapeutics [12]. The continuing search for new anticancer compounds in plant medicines and traditional foods is a realistic and promising strategy for its prevention [13]. Numerous groups with antitumor properties are plant derived natural products including alkaloids, phenylpropanoids, and terpenoids [14, 15]. The present study was conducted to evaluate the antiproliferative activity of fractionated extracts from some Jordanian plants. The plants were selected depending on the information gathered from traditional healers, reputed informants and their inclusion in Jordan *materia medica* as of probable antitumor activity [10].

## Results and Discussion

This study evaluated the antiproliferative potential of 44 extracts from 16 Jordanian plants (Table 1), prescribed mostly by traditional healers for cancer ailments [7, 10]. The ethanol extract of five plants exhibited high antiproliferative potential against the tested cell lines with some significant differences in selectivity (p < 0.05). These plants are *Ononis hirta* (aerial parts), *Ononis sicula* (aerial parts), *Verbascum sinaiticum* (flowers), *Inula viscosa* (flowers), and *Salvia pinardi* (aerial parts) (Table 2). These plants are studied for the first time for their antiproliferative activities except some *Inula* species [16, 17]. In the present study *Verbascum sinaiticum* flowers were more active than its aerial parts extracts (Table 2) which is in accordance with previous work that reported the superior activity of flowers of different *Verbascum* species compared with their aerial parts [18].

Further testing of the five active plants extracts showed that the extracts derived from chloroform fraction were more active than aqueous and butanol fraction (Table 3). This may indicate that the non polar active principles are responsible for the antiproliferative activity in these plants.

This result agrees with many previous researches that reported the bioactivity of non polar principles in plants like *Achillea santolina*, *Typhonium flagelliforme*, *Schisandra sphenanthera*, and *Scutellaria barbata* [17, 19–21].

**Fig.1.**
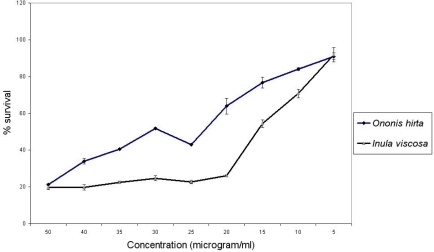
Antiproliferative activity of *Ononis hirta* (aerial parts) and *Inula viscosa* (flowers) aqueous methanol fraction against MCF-7 cell line.

According to the American National Cancer Institute (NCI), the criteria of cytotoxic activity for the crude extracts is an IC_50_ < 30 μg/ml [22]. The methanol fraction of *Ononis hirta* (aerial parts) and *Inula viscosa* (flowers) which exhibited the highest antiproliferative potential among the five active plants with IC_50_ values of 27.96 and 15.78 μg/ml respectively (Figure 1) fall within the NCI criteria, thus are considered as of promising anticancer potential. Previous studies reported the antiproliferative activity of *Inula graveolens*, *Inula helenium* and *Inula cappa* [16, 17, 23].

Although 14 different compounds were isolated from *Inula viscosa* whole plant [24], none was tested as anticancer agent and no data are available about the antiproliferative activity of *Inula viscosa* flowers. In our study the extracts of flowers of *Inula viscosa* showed promising activity (Table 4) which necessitates further isolation and identification of the active ingredients.

As for *Ononis hirta*, this is the first study to report its antiproliferative activity; the methanol extract of *Ononis hirta* (aerial parts) inhibited the proliferation of different cancer cell lines and showed selective toxicity (p < 0.05) toward MCF-7 cell line (IC_50_ values of 27.96, 54.22, and 41.87 μg/ml against MCF-7, Hep-2, and Vero cell lines) (Table 4). Fractions of some plants exhibited different activity on different cell lines. For example, the IC_50_ values of *n*-hexane fraction of *Ononis hirta* (aerial parts) were 72.06 for MCF-7 and 90.30 for Hep-2 (Table 4). This selectivity could be due to the sensitivity of the cell line to the active compounds in the extract or to tissue specific response [25].

Previous studies demonstrated the general bioactivity of *Ononis hirta* and *Inula viscosa* but not the anticancer activity [26]. It seems that the antiproliferative activities of such plant extracts against human cancer cell lines is novel and work along this line may be strongly correlated with plants use against a wide variety of afflictions.

**Fig. 2.**
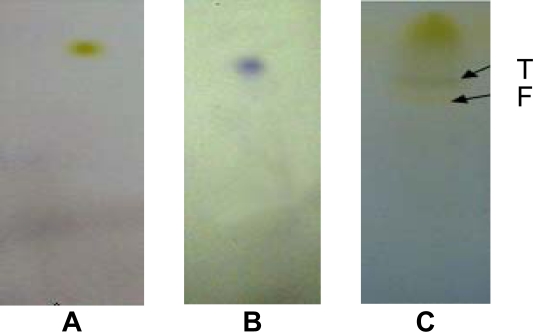
TLC fingerprint of *Ononis hirta*. (A) flavonoids positive control; (B) terpenoids positive control; (C) TLC fingerprint of *Ononis hirta* ethanol extract. Arrows show the presence of flavonoids (F) and terpenoids (T).

The association between flavonoids and reduced cancer risk has been reported in previous studies that showed a decrease in cancer risk with consumption of vegetables and fruits rich with flavonoids [27, 15]. The results of this study are in accordance with this finding since the phytochemical screening showed the presence of flavonoids in all active extracts (Table 5). While the presence of alkaloids with flavonoids (Figure 2) in *Ononis hirta* (aerial parts) extract may explain its superior activity compared with other plants studied in this work. The antiproliferative activity of total flavonoids and alkaloids isolated from different plants were reported [28, 15].

**Fig. 3.**
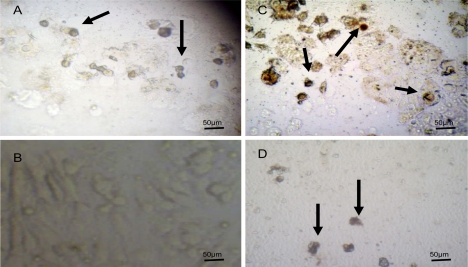
MCF-7 cells assayed by DeadEnd™ colorimetric TUNEL system to indicate cell apoptosis. (A) Positive control; (B) Negative control; (C) Cells treated with 25μg/ml of *Ononis hirta* methanol extract. (D) Cells treated with 25μg/ml *Inula viscosa* (flowers) methanol extract observed under the light microscope. Arrows show dark stained nuclei which indicate DNA fragmentation and nuclear condensation

The significant activity of methanol extract of *Ononis hirta* (aerial parts) and *Inula viscosa* (flowers) are thought to be a result of the induction of cell death by apoptosis as our results indicate (Figure 3). Park *et al*., [15] claimed that flavonoids would induce apoptosis in cancer cells. Reed and Pellecchia, [12] work in this direction stressed on inducing apoptosis as a desired strategy of controlling cancers. Our apoptotic test results would support the previous studies especially DNA fragmentation, nuclear condensation, and cell shrinkage which were clearly observed (Figure 3). This is not an exception since many commercially available chemotherapeutic agents and folk medicinal plants exert their anticancer effect by inducing cell apoptosis [21, 13]. However, this needs further comprehensive studies to be fully substantiated.

## Experimental

### Plant material

Plants were collected from Amman and Ajloun area in Jordan. The taxonomic identity of each plant was authenticated by Prof. Ahmad EL-Oqlah (Department of Biological Sciences, Yarmouk University, Irbid, Jordan) and Prof. Dawud EL-Eisawi (Department of Biological Sciences, University of Jordan, Amman, Jordan). Voucher specimens were deposited in the Department of Biological Sciences, University of Jordan, Amman, Jordan (Table 1).

### Plant extraction and fractionation

Different plant samples were dried at room temperature and were finely ground. Suitable amounts of the powdered plant materials were soaked in 95% ethanol (1L per 100g) for two weeks. The crude ethanol extracts were obtained after the solvent was evaporated at 40°C to dryness under reduced pressure using rotary evaporator (Buchi R-215, Switzerland). The residues were further subjected to solvent-solvent partitioning between chloroform and water. The dried chloroform extract was also partitioned between *n*-hexane and 10% aqueous methanol while butanol extract was fractionated from aqueous extract [6]. All solvents were evaporated to dryness under reduced pressure to produce the crude extracts which were collected and stored at −20°C for further testing.

### Cell lines and culture conditions

Hep-2 (larynx carcinoma), MCF7 (breast epithelial adenocarcinoma), and Vero (African green monkey kidney) cell lines were kindly provided by Dr. Mona Hassuneh (Department of Biological Sciences, University of Jordan). Cells were grown in Minimum Essential Medium Eagle (Gibco, UK) supplemented with 10% heat inactivated fetal bovine serum (Gibco, UK), 29 μg/ml L-glutamine, and 40 µg/ml Gentamicin. Cells were incubated in a humidified atmosphere of 5% CO_2_ at 37°C.

### Antiproliferative activity assay

The antiproliferative activity of plant extracts was measured using MTT (3-(4,5-dimethyl-thiazol-2-yl)-2,5-diphenyltetrazolium bromide) assay (Promega, USA). The assay detects the reduction of MTT by mitochondrial dehydrogenase to blue formazan product, which reflects the normal function of mitochondria and cell viability [29].

Exponentially growing cells were washed and seeded at 17000 cells/well (in 200 μl of growth medium) in 96 well microplates (Nunc, Denmark). After 24 h incubation, a partial monolayer was formed then the media was removed and 200 μl of the medium containing the plant extract (initially dissolved in DMSO) were added and re-incubated for 48 h. Then 100 μl of the medium were aspirated and 15 μl of the MTT solution were added to the remaining medium (100 μl) in each well. After 4 h contact with the MTT solution, blue crystals were formed. 100 μl of the stop solution were added and incubated further for 1h. Reduced MTT was assayed at 550 nm using a microplate reader (Das, Italy). Control groups received the same amount of DMSO (0.1%).Untreated cells were used as a negative control while, cells treated with vincristine sulfate were used as a positive control at the following concentrations 0.05, 0.1, 0.5, 1, 5, 10, 25, 50, 100 nM.

The MTT assay was undertaken in three stages. In the first stage, 100 μg/ml of the ethanol extract of each plant were tested against the cancer cell lines (Hep-2 and MCF-7). All plants that cause more than 50% inhibition of proliferation on any cell line were selected for further investigations in stage 2.

In the second stage, 100 μg/ml of each fraction (chloroform, *n*-hexane, butanol, aqueous, and methanol) of active plants were tested against the three cell lines (Hep-2, McF-7, and Vero). Fractions that showed more than 50% inhibition were selected for IC_50_ determination in stage 3.

In the third stage, eight concentrations (200, 100, 150, 100, 50, 25, 15, 5 μg/ml) were prepared from each active fraction and tested against the three cell lines. IC_50_ values were calculated as the concentrations that show 50% inhibition of proliferation on any tested cell line.

Stock solutions of the plant extract were dissolved in (DMSO) then diluted with the medium and sterilized using 0.2 μm membrane filters. The final dilution of extracts used for treating the cells contained not more than 0.1% DMSO. Data were reported as the average of three replicates. The antiproliferative effect of the tested extracts was determined by comparing the optical density of the treated cells against the optical density of the control (untreated cells).

### Phytochemical screening

Phytochemical screening using thin layer chromatography (TLC) was conducted for extracts that showed a potential antiproliferative activity against the tested cell lines.

Aliquots (50–75 μl) of the ethanol extract were applied 1cm from the base of the TLC plates (0.25 mm, Macherey-Nagel, Germany). Serial mixtures of chloroform and methanol (from 0–100 %) were used as eluents. Development of the chromatograms was done in a closed tank in which the atmosphere had been saturated with the eluent vapor by lining the tank with filter paper wetted with the eluent. For flavonoids and terpenoids identification, plates were sprayed with *p*-anisaldehyde/sulfuric acid reagent and carefully heated at 105°C for optimal color development [30]. For alkaloids detection, plates were sprayed with iodoplatinate acid and dried in the fume hood.

### Assessments of apoptosis in cell culture

Apoptosis was detected using terminal deoxynucleotidyl transferase (TdT) mediated-16-deoxyyuridine triphosphate (dUTP) Nick-End Labelling (TUNEL) system (Promega, USA). MCF-7 cells cultured in 24 well plates were treated with the methanol extracts of *Ononis hirta* (aerial parts) and *Inula viscosa* (flowers) at 25 μg/ml and incubated for 48 h. The assay was conducted according to the manufacturer’s instructions. Briefly, treated cells were fixed using 10% formalin followed by washing with phosphate buffer saline (PBS). Cells were then permeabilized using 0.2% triton X-100. Biotinylated dUTP in rTdT reaction mixture was added to label the fragmented DNA at 37°C for one hour, followed by blocking endogenous peroxidases using 0.3% hydrogen peroxide. Streptavidin HRP (1:500 in PBS) was added and incubated at room temperature for 30 minutes. Finally, hydrogen peroxide and chromagen diaminobenzidine were used to visualize nuclei with fragmented DNAs under the light microscope (Novex, Holland). Cells treated with 40 nM vincristine sulfate were used as a positive control while untreated cells were used as a negative control.

### Statistical analyses

The results are presented as means ± SEM of three independent experiments. Statistical differences among fractions were determined by one way ANOVA using Graph Pad Prism 5 (GraphPad Software Inc., San Diego, USA). Differences were considered significant at p < 0.05.

## Figures and Tables

**Tab. 1.** Ethnobotanical data of the studied plants

**Plant name, family, Voucher code**	**Local name**	**Parts used**	**Traditional and/or medicinal use**	**Mode of use**

*Populus alba* L., Salicaceae, MAHAS 2	Al-Hoor al-abya	Flowers	Depurative, tooth decay, skin lesions and herpes	Decoction, Infusion/oral
*Lavandula angustifolia* Mill., Lamiaceae, MAHAS 4	khuzama, lavender	Flowers	Bronchitis, cough, Antiseptic	Infusion/oral
*Jasminum sambac* Ait., Oleaceae, MAHAS 8	Yasmeen	Flowers	Ulceration, dermatosis, fever	Infusion
*Teucrium polium* L., Labiatae, MAHAS 9	Jeada	Aerial parts	Anti-inflammatory, Spasm, flatulence, diabetes, necrosis, kidney stones	Infusion
*Salvia pinardi* L., Labiatae, MAHAS 10	Miramia	Aerial parts	Sedative, for wound healing and herpes	Infusion
*Syringa vulg.* L., Oleaceae, MAHAS 11	Lailac	Aerial parts, seeds	Antihelminthic, anti-febrifuge, dry skin, treatment of malaria	Infusion, topical
*Phagnalon rupstre* L*.,* Asteraceae, MAHAS 12	Kadha	Aerial parts	Any disease of unknown reason, Inflammation, rheumatism, migraine, depression, scalp infection	Decoction, Infusion/oral
*Inula viscos*a (L.) Ait., Compositae, MAHAS 13	Taioon	Flowers	Antihelminthic, for lung cancer, Muscle relaxant	Decoction
*Luffa cylindrica* L., Araceae, MAHAS 14	Louf	Aerial parts	Treatment of cancer, post-delivery pain, inflammation, infections	Oral/ Infusion
*Pterocephalus pulverulentus* Boiss, Dipsacaceae, MAHAS 15	Abu Moch	Aerial parts	Ulceration	Gargling
*Mirabilis jalapa* L., Nyctaginaceae, MAHAS 16	Chap-Zarief	Aerial parts, roots, stems	Antiseptic, antiviral, Fungicide, Antiabortive, Useful in epilepsy and chronic bronchitis	Oral/ Infusion
*Narcissus tazetta* L., Amaryllidaceae, MAHAS 17	Narjes	Aerial parts, Flowers	Anticancer, Antiinflammatory, Memorigenic, and Sedative	Infusion
*Verbascum sinaiticum* L., Scrophulariaceae, MAHAS 18	Al-Omaya	Flowers, Aerial parts	Neural pain, herpes and bronchitis	Decoction
*Rosa damascena* Mill.; Rosaceae, MAHAS 19	Ward Demashqi	Receptacles, seeds	Antibacterial, treatment of cardiac diseases, colon cancer	Decoction
*Ononis sicula* Desf., Fabaceae, A O 298	Shibreq	Aerial parts	Skin cancer, lesions	Topical/wash
*Ononis hirta* L., Fabaceae, A O 297	Showk AL-Jamal	Aerial parts	Skin cancer, necrosis, herpes, cold sores	Mouth and skin wash

**Tab. 2.** The antiproliferative activity of 100 μg/ml of plant ethanol extracts against cancer cell lines. Plants in bold were selected for further evaluation. f: flower, a.p: aerial parts, s: seeds, r: receptacles.

**Plant**	**Yields (w:w %)**	**% Viability** ± **SEM**	
		**MCF-7**	**Hep-2**

*Teucrium polium* (a.p)	6.67	78 ± 4.51	58 ± 9.42
***Ononis hirta* (a.p)**	4.56	**17 ± 0.25**	**19 ± 2.01**
***Ononis sicula* (a.p)**	5.60	**40 ± 2.33**	53 ± 7.02
*Syringa vulgaris* (f)	5.60	71 ± 6.89	122 ± 10.50
*Syringa vulgaris* (s)	14.90	103 ± 1.57	152 ± 13.40
*Phagnalon rupstre* (a.p)	3.82	76 ± 1.53	118 ± 9.33
*Pterocephalus pulverulentus* (a.p)	8.19	80 ± 0.94	138 ± 16.39
*Lavandula angustifloia* (f)	10.36	96 ± 4.31	62 ± 0.70
*Jasminum sambac* (f)	29.00	82 ± 1.79	105 ± 15.33
*Luffa cylindica* (a.p)	11.55	83 ± 5.15	120 ± 15.56
*Mirabilis jalapa* (a.p)	18.40	60 ± 6.75	78 ± 9.98
*Populus alba* (f)	3.81	100 ± 1.85	100 ± 7.47
*Narcissus tazetta* (f)	18.80	60 ± 0.89	80 ± 5.07
*Narcissus tazetta* (a.p)	6.50	57 ± 0.87	72 ± 3.05
***Verbascum sinaiticum* (f)**	17.50	**40 ± 1.30**	66 ± 11.06
*Verbascum sinaiticum* (a.p)	14.60	60 ± 5.12	75 ± 10.33
*Rosa damascene* (r)	7.19	160 ± 7.46	143 ± 16.37
***Inula viscosa* (f)**	15.40	**30 ± 0.82**	67 ± 6.41
***Salvia pinardi*** (a.p)	4.30	**38 ± 6.11**	70 ± 5.95

**Tab. 3.** Antiproliferative activities of 100 μg/ml of plant extract active fractions on cell lines as measured by the MTT assay. Fractions in bold were selected for IC_50_ determination. ND: not determined.

**Plant**	**Fraction**	**% Viability** ± **SEM**		
		**MCF-7**	**Hep-2**	**Vero**

*Ononis hirta*	**Chloroform**	13.54 ± 0.34	12.03 ± 0.74	10.34 ± 0.27
	Aqueous	115.16 ± 0.84	129.58 ± 7.50	129.17 ± 2.54
	Butanol	100.66 ± 2.62	104.32 ± 0.98	115.07 ± 6.55
	***n*-hexane**	12.45 ± 0.24	9.55 ± 0.52	10.60 ± 0.64
	**Methanol**	14.01 ± 0.54	8.92 ± 0.33	8.38 ± 0.29

*Verbascum sinaiticum* (flower)	**Chloroform**	23.41 ± 1.61	ND	21.39 ± 0.97
	Aqueous	152.50 ± 9.63	ND	105.96 ± 3.85
	Butanol	120.16 ± 0.49	ND	77.01 ± 10.86
	***n*-hexane**	45.93 ± 2.82	ND	26.82 ± 4.38
	**Methanol**	25.15 ± 2.52	ND	17.68 ± 1.01

*Inula viscosa*	**Chloroform**	28.02 ± 2.94	ND	21.91 ± 0.65
	Aqueous	164.08 ± 21.10	ND	131.99 ± 5.34
	Butanol	73.45 ± 6.29	ND	47.55 ± 2.33
	***n*-hexane**	24.81 ± 2.23	ND	11.92 ± 0.51
	**Methanol**	48.68 ± 6.53	ND	40.87 ± 2.27

*Salvia pinardi*	**Chloroform**	45.47 ± 5.52	ND	44.78 ± 1.90
	Aqueous	108.33 ± 1.56	ND	153.01 ± 2.56
	Butanol	131.60 ± 2.49	ND	152.48 ± 4.99
	***n*-hexane**	35.38 ± 0.18	ND	38.40 ± 2.88
	**Methanol**	43.64 ± 2.32	ND	29.83 ± 1.18

*Ononis sicula*	**Chloroform**	27.27 ± 2.80	26.67 ± 1.26	26.16 ± 3.17
	Aqueous	111.58 ± 1.81	123.33 ± 3.76	116.98 ± 5.62
	Butanol	113.19 ± 0.42	124.04 ± 9.35	162.90 ± 7.50
	***n*-hexane**	55.60 ± 2.25	41.92 ± 1.84	61.96 ± 6.09
	Methanol	118.18 ± 7.04	140.61 ± 5.14	123.00 ± 21.13

**Tab. 4.** IC_50_ determination of the most active fractions of plants tested. ND: not determined.

**Plant**	**Fraction**	**IC_50_ value (μg/ml) ± SEM**		
		**MCF-7**	**Hep-2**	**Vero**

*Ononis hirta*	Chloroform	44.58 ± 1.42	48.75 ± 1.84	42.74 ± 2.18
	*n*-hexane	72.06 ± 2.79	90.30 ± 2.02	86.60 ± 0.59
	Methanol	27.96 ± 0.54	54.22 ± 3.03	41.87 ± 2.72

*Ononis sicula*	Chloroform	66.02 ± 1.58	75.33 ± 2.23	79.51 ± 4.38
	*n*-hexane	114.11 ± 2.42	93.34 ± 0.91	122.72 ± 2.46

*Verbascum sinaiticum* (flower)	Chloroform	87.30 ± 0.80	ND	79.62 ± 2.21
	*n*-hexane	95.43 ± 6.40	ND	130.76 ± 5.50
	Methanol	119.04 ± 1.94	ND	188.19 ± 1.08

*Inula viscosa*	Chloroform	77.97 ± 0.89	ND	44.82 ± 2.33
	*n*-hexane	166.65 ± 0.08	ND	188.52 ± 2.34
	Methanol	15.78 ± 0.59	ND	79.33 ± 3.37

*Salvia pinardi*	Chloroform	94.78 ± 4.42	ND	97.03 ± 1.67
	*n*-hexane	192.28 ± 3.80	ND	190.37 ± 9.20
	Methanol	85.49 ± 2.54	ND	97.08 ± 5.62

**Tab. 5.** Thin layer chromatography analysis of the most active plants extracts

**Plant**	**Alkaloids**	**Flavonoids**	**Terpenoids**

*Ononis hirta* (aerial parts)	+	+	+
*Verbascum sinaiticum* (flowers)	−	+	+
*Inula viscose* (flowers)	−	+	+
*Salvia pinardi* (aerial parts)	−	+	+
